# The DNA polymerase activity of Pol ε holoenzyme is required for rapid and efficient chromosomal DNA replication in *Xenopus *egg extracts

**DOI:** 10.1186/1471-2091-7-21

**Published:** 2006-08-22

**Authors:** Koh Shikata, Taro Sasa-Masuda, Yukiko Okuno, Shou Waga, Akio Sugino

**Affiliations:** 1 Laboratories for Biomolecular Networks, Graduate School of Frontier Biosciences, Osaka University, 1–3 Yamada-oka, Suita, Osaka 565-0871, Japan; 2 Department of Biology, Graduate School of Science, Osaka University, Toyonaka, Osaka 560-0043, Japan; 3 Research Institute, Thermostable Enzyme Laboratory Co., Ltd, 1-8-31 Midoriga-oka, Ikeda, Osaka 563-8577, Japan; 4 Braun Laboratories 147-75, California Institute of Technology, Pasadena, California 91125, USA; 5 Department of Biology, Graduate School of Science, Osaka University, Toyonaka, Osaka 560-0043, Japan

## Abstract

**Background:**

DNA polymerase ε (Pol ε) is involved in DNA replication, repair, and cell-cycle checkpoint control in eukaryotic cells. Although the roles of replicative Pol α and Pol δ in chromosomal DNA replication are relatively well understood and well documented, the precise role of Pol ε in chromosomal DNA replication is not well understood.

**Results:**

This study uses a *Xenopus *egg extract DNA replication system to further elucidate the replicative role(s) played by Pol ε. Previous studies show that the initiation timing and elongation of chromosomal DNA replication are markedly impaired in Pol ε-depleted *Xenopus *egg extracts, with reduced accumulation of replicative intermediates and products. This study shows that normal replication is restored by addition of Pol ε holoenzyme to Pol ε-depleted extracts, but not by addition of polymerase-deficient forms of Pol ε, including polymerase point or deletion mutants or incomplete enzyme complexes. Evidence is also provided that Pol ε holoenzyme interacts directly with GINS, Cdc45p and Cut5p, each of which plays an important role in initiation of chromosomal DNA replication in eukaryotic cells.

**Conclusion:**

These results indicate that the DNA polymerase activity of Pol ε holoenzyme plays an essential role in normal chromosomal DNA replication in *Xenopus *egg extracts. These are the first biochemical data to show the DNA polymerase activity of Pol ε holoenzyme is essential for chromosomal DNA replication in higher eukaryotes, unlike in yeasts.

## Background

Three structurally and functionally distinct DNA polymerases, known as DNA polymerases α, δ, and ε (Pol α,-δ, and -ε, respectively), are required for chromosomal DNA replication in yeasts [1-3]. The complex structure of each Pol α, -δ, and -ε is well conserved from yeast to human [4], thus their function inside the cell is also believed to be conserved [4].

DNA primase initiates DNA replication by synthesizing a short oligo-ribonucleotide primer which is immediately elongated by Pol α to form short RNA-DNA fragments on both leading and lagging strand of DNA. Pol δ elongates the short RNA-DNA fragment initiated by Pol α-primase to make a mature Okazaki fragment [2,5]. To carry out processive DNA synthesis *in vitro*, Pol δ requires PCNA and its loader, Replication Factor-C (RF-C) [5]. In cooperation with Fen1p (Rad27p), Dna2p and RPA, Pol δ also plays a crucial role in processing RNA-linked Okazaki-fragments in budding yeast [5].

Although the precise role of Pol ε *in vivo *is still unclear, it has been implicated in DNA replication, repair, recombination, and mitosis [1,2,4]. Pol ε has at least one essential function in both budding and fission yeasts [3,6], and several lines of evidence suggest that Pol ε plays an essential catalytic role during chromosomal DNA replication. Yeast cells harboring a temperature-sensitive *pol2 *allele are temperature sensitive for growth and express a thermolabile Pol ε DNA polymerase activity [7]. Furthermore, Pol2p is associated with replication forks during S phase [8] and *pol2 *mutants fail to complete chromosomal DNA replication [7,9]. Furthermore, 3'–5' exonuclease-deficient mutants of *POL2 *and *POL3 *(Pol δ) accumulate strand-specific lesions in chromosomal DNA [10-13]. These observations support models for chromosomal DNA replication in which Pol ε and Pol δ play leading strand- and lagging strand-specific roles during chromosomal DNA replication, respectively. Pol ε has been proposed as the leading strand DNA polymerase because Pol ε is a highly processive polymerase without PCNA [1,14] and *pol3 *mutants have defects in maturation of Okazaki fragments [5]. Nevertheless, it has been reported that the amino-terminal portion of budding yeast Pol ε (Pol2p), which includes motifs required for DNA polymerase and exonuclease activities, is dispensable for DNA replication, DNA repair, and viability [15]. However, this conclusion is controversial, because the polymerase active domain mutant (polymerase-dead-mutant) is lethal [16] and our studies showed that the deletion mutant confers temperature-sensitivity for growth, a defect in DNA elongation, premature senescence, and short telomeres. Furthermore, this pol2p deletion is lethal in combination with temperature-sensitive *cdc2 *and with exonuclease-deficient Pol δ (*pol3-01*). These results suggest that Pol ε plays a crucial role in maintaining genomic integrity [17,18].

In higher eukaryotes, the function of Pol ε is much less clear than in *S. cerevisiae*, although some reports showed that Pol ε is required for cellular chromosomal DNA replication [19-21]. In order to understand how Pol ε involves in higher eukaryotic chromosomal DNA replication, we have been characterizing DNA replication catalyzed by *Xenopus *egg extracts [22,23]. We have shown previously that both the initiation and elongation steps of chromosomal DNA replication are markedly impaired in Pol ε-depleted *Xenopus *egg extracts, resulting in significant reduction of the overall DNA synthesis as well as accumulation of small replication intermediates. Moreover, despite the decreased DNA synthesis, excess amounts of Pol α are loaded onto the chromatin template in Pol ε-depleted extracts, indicative of the failure of proper assembly of DNA synthesis machinery at the fork [22]. Although these experiments clearly demonstrate that Pol ε is required for a normal chromosomal DNA replication in *Xenopus *egg extracts, they did not give any answer whether DNA polymerase activity of Pol ε directly involves in chromosomal DNA replication process.

In the present report we show that the reconstituted and purified *Xenopus *Pol ε holoenzyme, which is proficient in DNA polymerase activity, fully complements DNA replication defect of Pol ε-depleted *Xenopus *egg extracts, but neither DNA polymerase domain deleted Pol ε holoenzyme, DNA polymerase-dead-Pol ε holoenzyme, the catalytic subunit of Pol ε, nor other subunits of Pol ε holoenzyme complements the defect at all. These results clearly prove that the DNA polymerase activity of Pol ε holoenzyme directly participates in chromosomal DNA replication in *Xenopus *egg extracts and that the products made by Pol ε-depleted *Xenopus *egg extracts are not fully replicated DNA.

## Results

### Isolation of cDNA encoding each subunit of *Xenopus *Pol ε holoenzyme

A previous study reported cloning of a partial cDNA for the catalytic subunit (p260) of *Xenopus *Pol ε (xPol ε) [24], and in this report, the corresponding full-length cDNA was cloned by 5' Rapid amplification of cDNA ends (5' Race) (see Additional file 1). The fact that the full length cDNA encodes a 2285 amino acid residue protein whose predicted amino acid sequence is 81% identical to human p260 [25] and 62% identical to *S. cerevisiae *Pol2p [6] within the polymerase domain (see Additional file 2 and Additional file 3) confirms the identity of the cloned fulllength cDNA. Full length cDNA encoding p17 and p12 of xPol ε were also cloned using 3' RACE, *Xenopus *ovary mRNA and partial cDNA clones 4783571(5') (GenBank ACC# BI349483) and PBX0037B02(5') (GenBank ACC# AW635703), respectively. The predicted amino acid sequences of p12 and p17 showed very high similarity to p12 and p17 of human Pol ε, respectively [26](see Additional file 4), confirming the identity of the cloned cDNAs. cDNA encoding p60 of xPol ε holoenzyme was cloned previously [22]; thus, with this report, full length cDNA clones have been isolated for all four subunits of xPol ε holoenzyme.

### The C-terminal Zinc finger domain of p260 is required for binding p60 but not p12-p17

**Figure 1:**
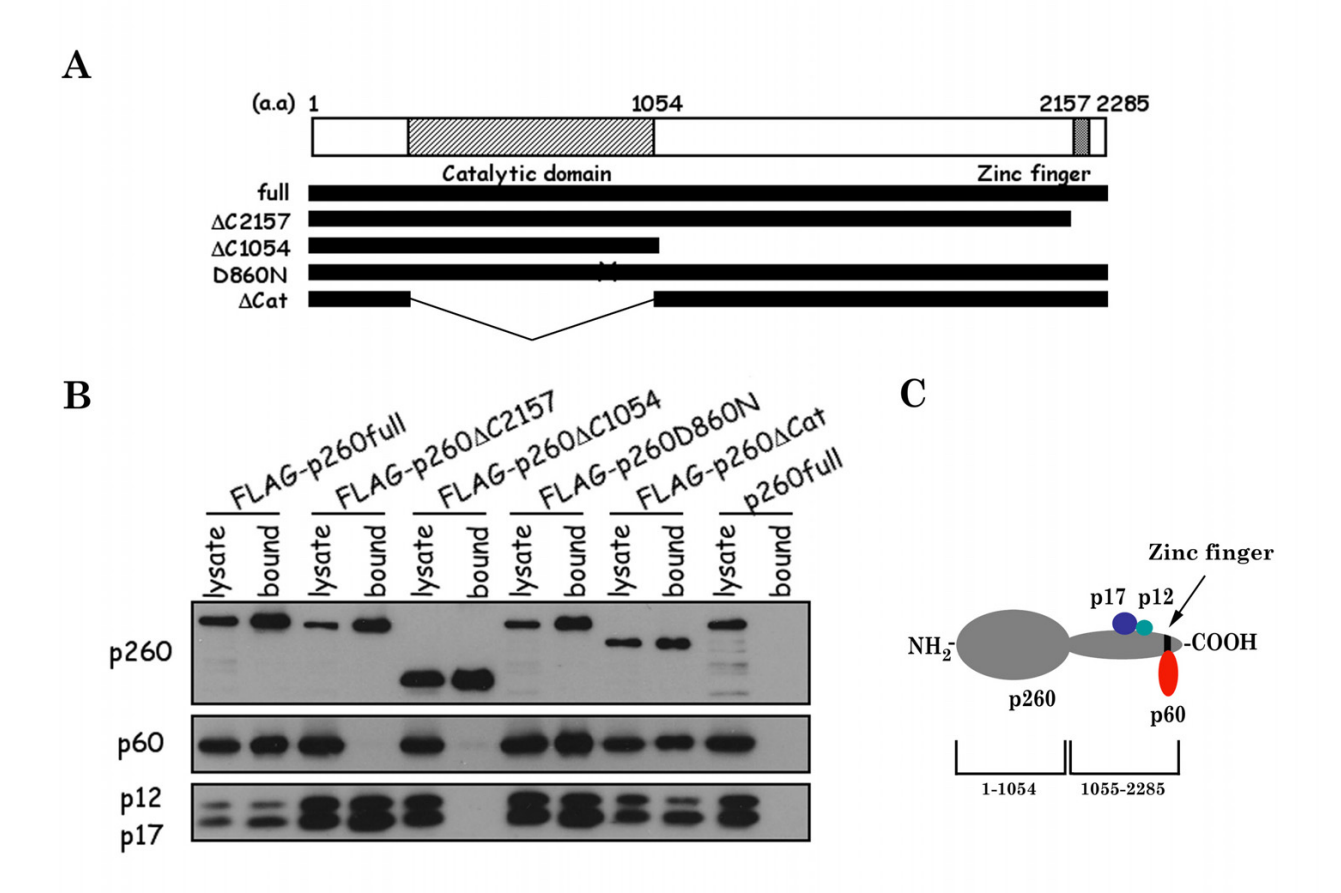
**p12, p17 and p60 of *Xenopus *Pol ε interact with the C-terminal region of p260**. (A) Schematic representation of wild type (full)- and mutant (ΔC2157, ΔC1054, Δ860N, and ΔCat) forms of xPol ε p260. The conserved catalytic DNA polymerase domain and putative zinc finger domain of Pol ε are indicated. (B) FLAG-tagged p260 was co-expressed with p60, p17 and p12 in insect cells. Cell lysates were prepared and immunoprecipitated with anti-FLAG antibody and the precipitates were subjected to SDS-PAGE followed by immunoblotting with antibodies for each subunit. "Lysates" and "bound" indicate total protein and immunoprecipitated proteins, respectively. (C) Schematic representation of *Xenopus *Pol ε holoenzyme.

**Figure 3:**
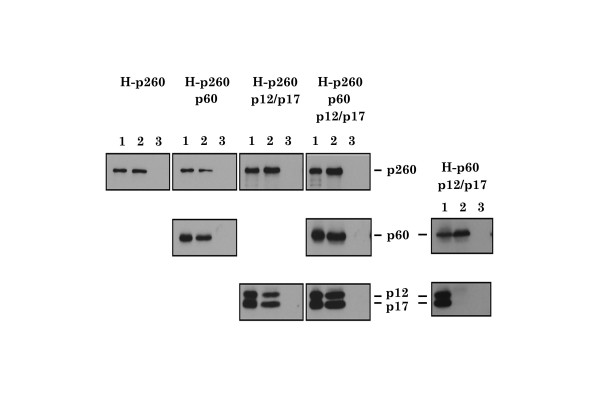
**Interactions between histidine-tagged p260 (H-p260) or p60 (H-p60) and other subunits of Pol ε**. Interactions between histidine-tagged p260 (H-p260) or p60 (H-p60) and other subunits of Pol ε were investigated using a pull down assay with Ni^2+ ^-chelating beads and crude extracts prepared from insect cells expressing the indicated subunits of xPol ε. Cell lysates containing H-p260 or H-p60 (lane 1) and bound proteins (lane 2) were subjected to SDS-PAGE, followed by immunoblotting with the indicated antibodies. Beads without Ni^2+ ^were also used as a control (lane 3).

Deletion mutants of xPol ε p260 were constructed and used to identify protein regions involved in the interfaces between p260 and the other subunits of Pol ε holoenzyme. For these experiments, p260 deletion mutants were fused to the FLAG epitope tag (Fig. 1A) and the following mutants were generated: (a) FLAG-p260full, (b) FLAG-p260ΔC2157, (c) FLAG-p260ΔC1054, (d) FLAG-p260D860N, and (e) FLAG-p260ΔCat (equivalent to yeast Pol2-16p [15,16,27]). The p260 mutants were co-expressed with xPol ε p60, p12 and p17 using a baculovirus insect cell expression system and immunoprecipitated with anti-Flag antibodies. Figure 1B shows that anti-Flag antibodies co-immunoprecipitated p260, p60, p12, and p17 from crude extracts of insect cells expressing wild type p260, p60, p12, and p17. In contrast, in cells expressing p260ΔC2157, p12 and p17 co-immunoprecipitated with the catalytic subunit but p60 did not, and in cells expressing p260ΔC1054, no other subunits of Pol ε holoenzyme co-immunoprecipitated with the p260 mutant protein. These results suggest that p60, but not p12 or p17, interacts with p260 residues 2157 to 2285, which includes Zinc-finger motifs (Fig. 1C), and that p12 and p17 interact with a motif or region between residues 1054 and 2157 of p260 (Fig. 1C). Both p260D860N (a DNA polymerase-dead-point mutant) and p260ΔCat did not interfere with formation of the xPol ε holoenzyme, because all four Pol ε subunits co-immunoprecipitated from cells expressing these p260 mutants. In cells expressing histidine-tagged p60 (H-p60), p12, and p17, Ni^2+ ^-chelating beads only precipitated H-p60, indicating that H-p60 does not interact directly with p12 or p17, or both subunits (Fig. 3), although it was known that both subunits interact each other and form a 1:1 complex in insect cells (data not shown) as *S. cerevisiae *Dpb3p-Dpb4p complex [28].

### DNA polymerase activity of xPol ε holoenzyme is required for DNA synthesis in *Xenopus *egg extracts

**Figure 2:**
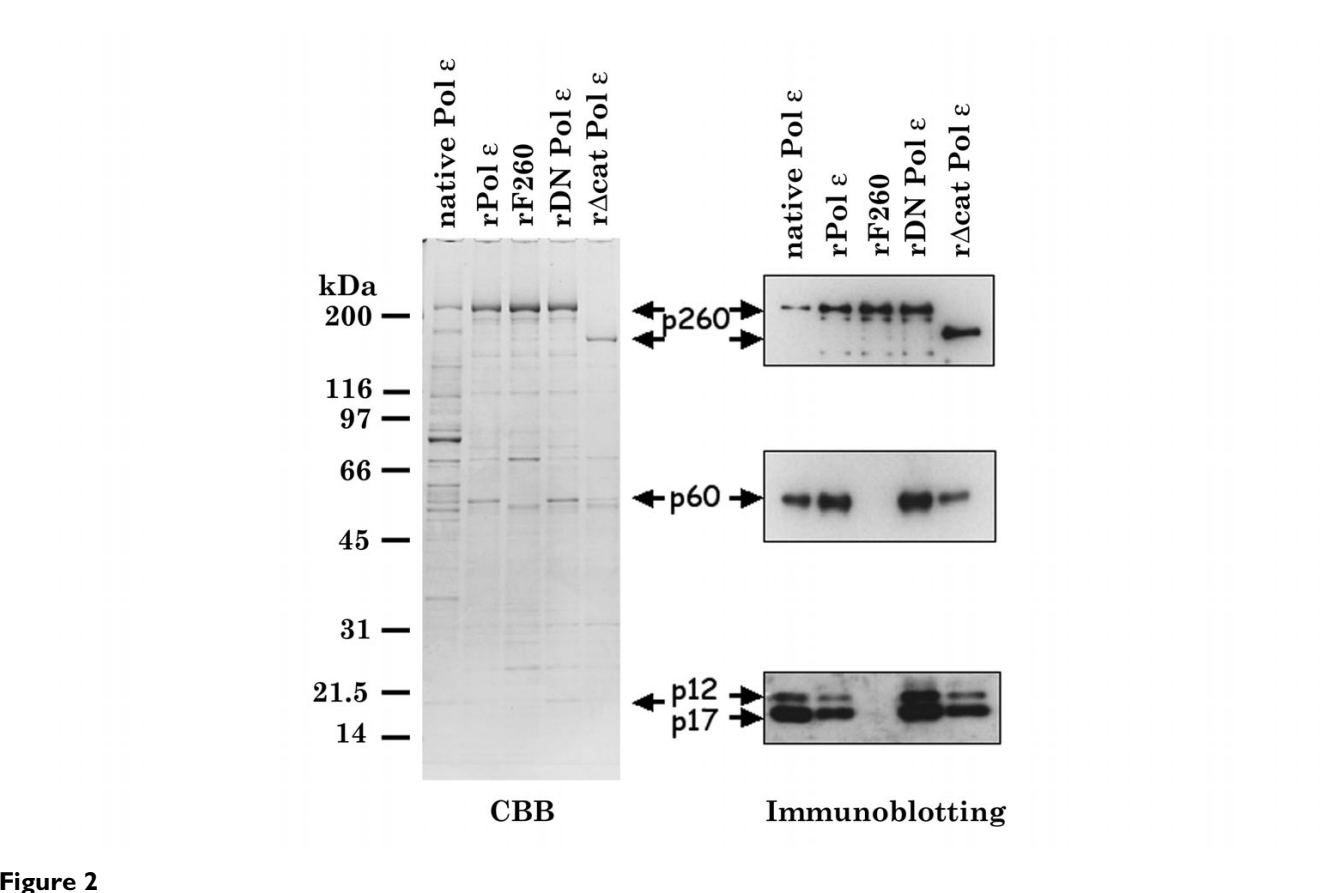
**Purified xPol ε holoenzyme from insect cells**. Wild type and mutant FLAG-tagged p260 was co-expressed with p60, p17 and p12 in insect cells and Pol ε complexes were purified by DEAE Sepharose and anti-FLAG antibody chromatography. Fractions were eluted from the antibody affinity column, pooled and subjected to SDS-PAGE followed by CBB staining (Left) and immunoblotting (Right). Native *Xenopus *Pol ε (native) holoenzyme was purified from *Xenopus *egg extracts as described previously [22]. Fractions shown are rPol ε, rDN Pol ε, rΔcat Pol ε, and rF260 (see text). rF260 is FLAG-tagged p260.

Previous studies suggested that the amino-terminal portion of yeast Pol ε is dispensable for DNA replication, DNA repair, and viability. Because this portion of the enzyme includes all known DNA polymerase and exonuclease motifs, these results suggest that the DNA polymerase activity of Pol ε is dispensable for chromosomal DNA replication in yeast [15,16,27]. To investigate this possibility further, the xPol ε mutant complexes described in Figure 2 as well as partial holoenzyme complexes lacking specific subunits were purified from insect cells and used in an *in vitro Xenopus *chromosomal DNA replication system with Pol ε-depleted *Xenopus *egg extracts. The following xPol ε holoenzyme complexes were used: (a) wild type r-xPol ε holoenzyme, (b) Pol ε-DN containing p260D860N, (c) Pol ε ΔCat containing p260ΔCat, (d) p260p60 sub-complex of xPol ε, (e) p260-p12-p17 sub-complex of xPol ε, and (f) p260 (Fig. 2 and 3). The specific activity of each enzyme complex is summarized in Table 1. Although we did not measure the specific activity of p260-p60 and p260-p12-p17 sub-complexes, they were similarly purified as r-xPol ε holoenzyme and p260 and might be the same specific activity as that of p260 alone. Pol ε-DN and Pol ε ΔCat holoenzymes do not have any significant DNA polymerase activity as predicted, although a slight contaminating DNA polymerase activity could be detected (Table 1). This may be due to contamination of insect DNA polymerase activity. It should be noted here that recombinant xPol ε holoenzyme complexes (r-xPol ε) purified from insect cells had minor contaminants, and all enzyme preparations except Flag-tagged p260 included p60, p12, and p17 in amounts that were detected by immunoblotting.

**Table 1:** The specific activity of the DNA polymerase

**Various form of xPol ε**	**the specific activity (units/mg protein)**
Native xPol ε (n-xPol ε) holoenzyme	4,800,000
Wild type r-xPol ε holoenzyme	530,000
Pol ε-DN containing p260D860N	2200
Pol ε ΔCat containing p260ΔCat	1100
p260/p60 sub-complex of xPol ε	Not determined
p260/p12/p17 sub-complex of xPol ε	Not determined
p260 alone	500,000

**Figure 4:**
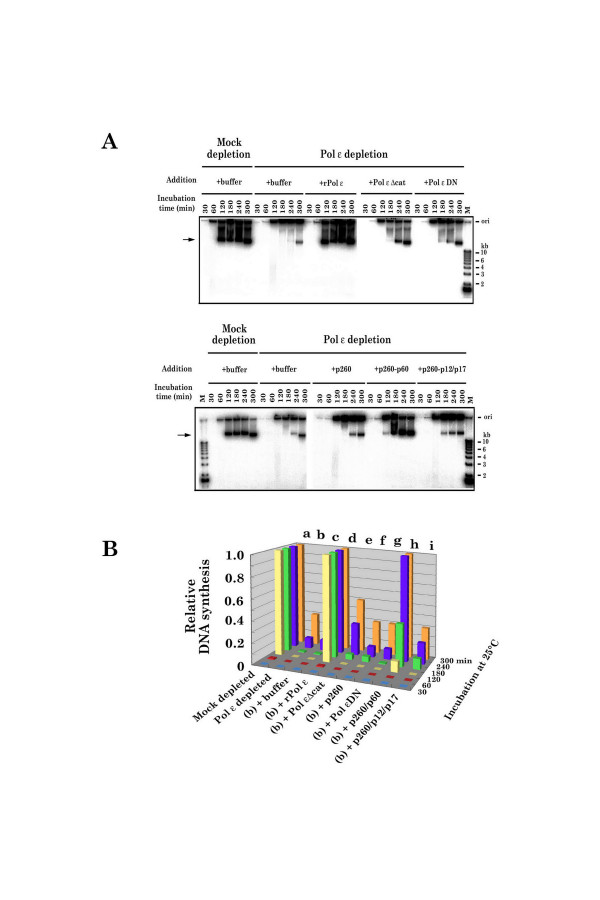
**DNA replication activity in Pol ε-depleted *Xenopus *egg extracts**. (A) The same number of wild type-, mutant or partial r-xPol ε complex molecule, which has been estimated based on the result of Fig. 2, was added to xPol ε-depleted egg extracts as indicated (see text for detailed description of mutants and partial complexes). DNA replication was initiated by the addition of sperm chromatin and the rate and extent of DNA synthesis was measured by neutral agarose gel electrophoresis followed by autoradiography [22]. The origin (well) is and DNA size markers are indicated on the right. (B) Radioactive material, which migrated slower than the 23-kb marker DNA (shown by an arrow in (A)), was considered to be fully replicated product and quantified by a scintillation counter.

Figure 4 shows that the extent and rate of DNA synthesis were greatly reduced in Pol ε-depleted *Xenopus *extracts, and this defect was fully complemented by addition of purified native (n-xPol ε)(data not shown, and [22]) or wild type recombinant xPol ε (r-xPol ε). In contrast, addition of xPol ε ΔCat or xPol ε DN to Pol ε-depleted *Xenopus *extracts only partially (30–40%) complemented the replication defect and did not restore normal kinetics of the initiation of DNA replication (Fig. 4). Furthermore, neither p260, p260-p12-p17 nor p12-p17 complemented the defect in DNA synthesis in xPol ε-depleted *Xenopus *extracts. Of the partial holoenzyme complexes tested, only the p260-p60 sub-complex of xPol ε significantly complemented the defect (Fig. 4). These results clearly demonstrate that the DNA polymerase activity of xPol ε holoenzyme is required for normal, rapid and efficient chromosomal DNA replication in *Xenopus *egg extracts.

### xPol ε holoenzyme interacts with xCdc45p, xCut5p, and xGINS

**Figure 5:**
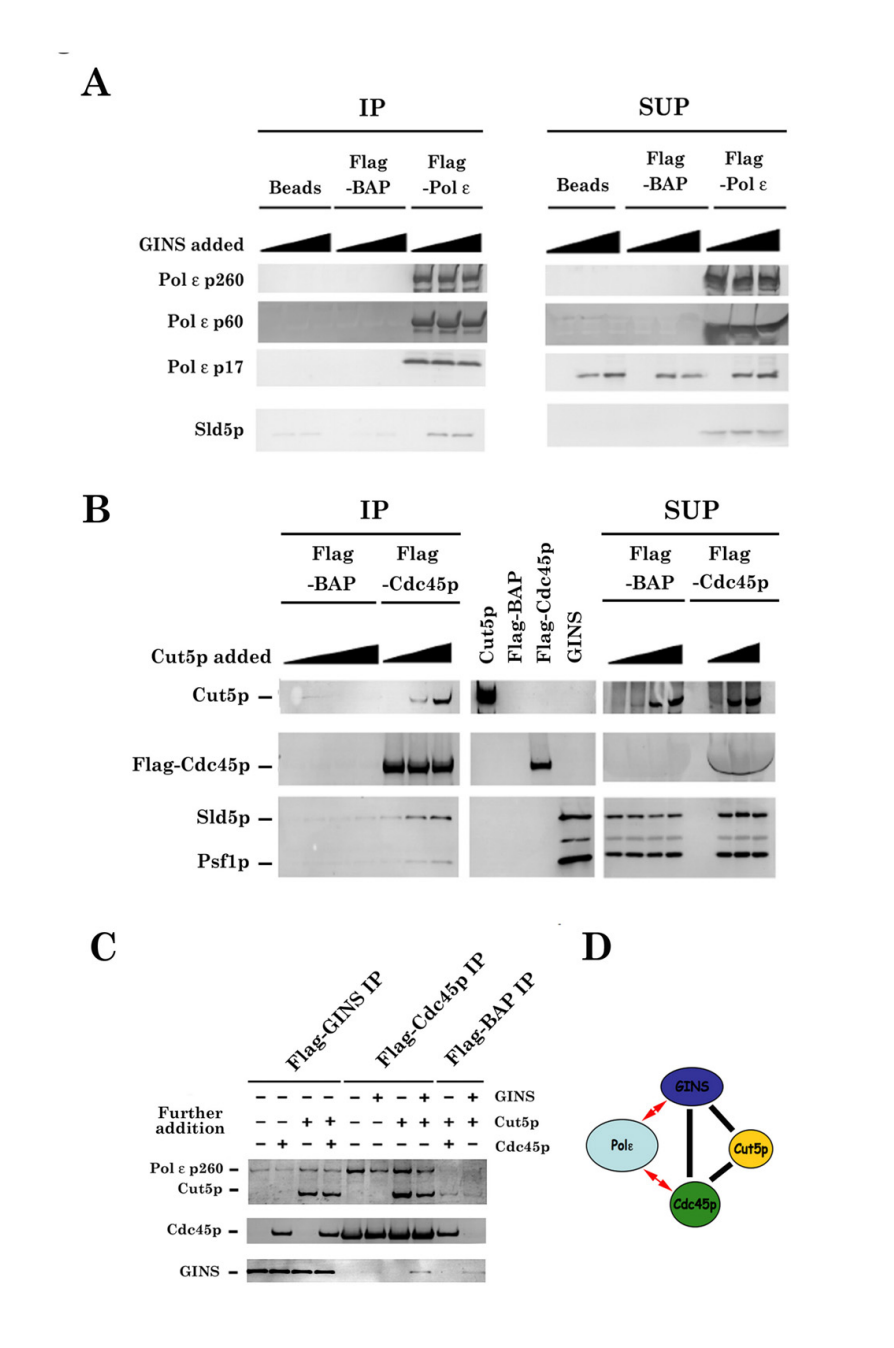
***Xenopus *Pol ε holoenzyme interacts directly with xGINS, xCdc45p, and xCut5p *in vitro***. (A) Flag-tagged r-xPol ε holoenzyme was incubated with xGINS [29] for 1 h at 4°C and immunoprecipitated with anti-Flag antibody. The immunoprecipitates were analyzed by SDS-PAGE followed by Western blotting. IP; immunoprecipitates, SUP; supernatant. (B) Increasing concentrations of xCut5p were incubated with xGINS and Flag-tagged xCdc45 [30] or Flag-tagged BAP for 1 h at 4°C and immunoprecipitated as in (A). (C) r-xPol ε holoenzyme was pre-incubated with either Flag-tagged xGINS or Flag-tagged xCdc45p prior to addition of the indicated protein(s). Reactions were analyzed as in (A). (D) A model for the interaction between r-xPol ε and replication accessory proteins (xGINS, xCdc45, and xCut5).

Previous studies suggested that Cdc45p, Cut5p, and GINS are critical for loading Pol α and Pol ε onto DNA replication origins and for initiating chromosomal DNA replication in yeast and *Xenopus *egg extracts [2,22,24,29-31]. However, those studies did not show any direct interaction between these initiation proteins and Pol ε holoenzyme. Therefore, in this study, we tested this possibility by measuring physical interactions between r-xPol ε holoenzyme and xCdc45p, xCut5p, and xGINS. The experiments were performed by incubating r-xPol ε holoenzyme with these proteins and immunoprecipitating protein complexes with anti-FLAG antibody. Figure 5A shows that xGINS was co-immunoprecipitated with Pol ε holoenzyme from reactions containing r-xPol ε holoenzyme and recombinant xGINS [29]. Addition of xCdc45p, but not xCut5p [31], slightly disrupted the complex between r-xPol ε holoenzyme and xGINS (Fig 5C), and incubation of xCdc45, xCut5p, and xGINS in the absence of r-xPol ε holoenzyme resulted in formation of a heterotrimeric complex lacking xPol ε holoenzyme (Fig. 5B). xCdc45p also interacts directly with xPol ε holoenzyme *in vitro *(Fig. 5C), and the amount of this complex decreased slightly in the presence of xGINS, but not in the presence of xCut5p (Fig. 5C). These results suggest that the trimeric xCdc45/xCut5p/xGINS complex interacts with xPol ε holoenzyme, and this interaction may involve direct contacts with xCdc45p and xGINS but not with xCut5p (Fig. 5D).

### xGINS stimulates xPol ε-holoenzyme-catalyzing DNA synthesis

**Figure 6:**
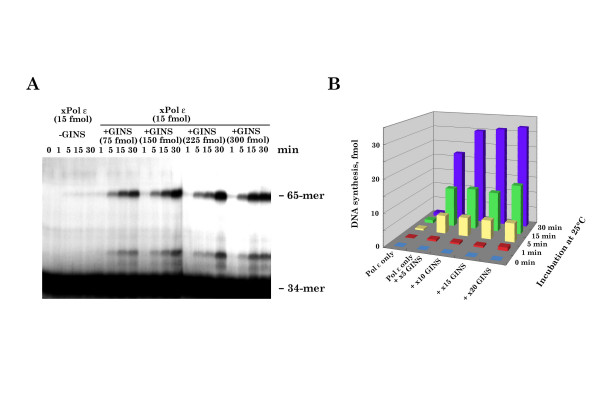
**xGINS stimulates DNA synthesis by xPol ε holoenzyme**. (A) DNA synthesis reactions (10 μl) contained 200 fmol ^32^P-labeled 34-mer primer/65-mer template replication substrate [32], 15 fmol r-xPol ε holoenzyme and 45 fmol (×5), 150 fmol (×10), or 300 fmol (×20) xGINS as indicated. Reactions were incubated at 25°C for the indicated amount of time, terminated by addition of stop solution (5 μl), and analyzed by sequencing gel and autoradiography [32]. The ^32^P-labeled 65-mer is the reaction product and the ^32^P-labeled 34-mer is the primer.(B) The amount of the reaction products in (A) (65-mer) was quantified by Image analyzer (Fiji).

Previous studies demonstrated a direct interaction between yeast GINS and yeast Pol ε holoenzyme and that this interaction stimulates the rate and processivity of DNA synthesis by Pol ε holoenzyme [32]. A similar result was obtained using r-xPol ε and r-xGINS [29]. As observed previously for yeast GINS, maximum stimulation by xGINS was obtained at a stoichimetry of 5–10 xGINS/xPol ε (Fig. 6). Under these conditions, however, xCdc45p or xCdc45p plus xCut5p did not stimulate the rate or extent of DNA synthesis by r-xPol ε holoenzyme (see Additional file 5).

## Discussion

Previous genetic studies in yeast suggested that Pol ε plays an important role during chromosomal DNA replication [1,7-9]. However, because the amino-terminal portion of Pol ε, that is required for its DNA polymerase- and exonuclease activities, is dispensable for yeast DNA replication, repair, and viability [15,16,27], the role of Pol ε during DNA replication has remained obscure. This study explores this role using an *in vitro Xenopus *DNA replication system and wild type and mutant forms of r-xPol ε holoenzyme. Here we show that the DNA replication defect in xPol ε-depleted *Xenopus *egg extracts is readily corrected by native (n-xPol ε) (data not shown and [22]) or recombinant xPol ε (r-xPol ε) holoenzyme or the p260-p60 Pol ε sub-complex, but not by p260ΔCat holoenzyme, p260 DN, p260 or p260-p12-p17 (Fig. 4). Because the former enzymes are polymerase proficient, while p260ΔCat holoenzyme and p260 DN are polymerase-deficient, although these preparations contained a small amount of DNA polymerase activity, and the last two sub-complexes do not contain the second essential subunit of xPol ε holoenzyme (Table 1), these results clearly demonstrate that the DNA polymerase activity of Pol ε holoenzyme is required for chromosomal DNA replication in *Xenopus *egg extracts. Note that both p260ΔCat and p260 DN holoenzymes partially complements DNA replication defect of xPol ε-depleted egg extracts (Fig. 4). However, these DNA synthesis activities never reach to the levels of either mock-depleted-, n-xPol ε holoenzyme-, or r-xPol ε holoenzyme-supplemented egg extracts even after long time incubation (Fig. 4 and [22]), thus we conclude that those residual activity of DNA synthesis upon addition of catalytically dead polymerases is not significant to the key issue of this paper.

We do not know why n-xPol ε holoenzyme preparations always exhibit higher specific activity of DNA polymerase than that of r-xPol ε holoenzyme (Table 1), although both polymerase holoenzymes equally complement the defect of the Pol ε-depleted *Xenopus *egg extracts ([22] and Fig. 4). However, we noticed that the reconstituted r-xPol ε holoenzyme is much more volatile than n-xPol ε holoenzyme during purification (Shikata K and Sugino A, unpublished results). Thus, it is possible that r-xPol ε holoenzyme purified from insect cells would not be fully activated yet and it might be activated during incubation with xPol ε-depleted egg extracts by unknown mechanism(s) and function in the egg extracts as does n-xPol ε holoenzyme.

The previous studies showed that a yeast strain consisting of a deletion of the amino-terminal portion of Pol2p (*pol2-16*) is temperature-sensitive for its growth, has a defect in elongation of DNA replication, has a short cell senescence, has a shorting telomere length, and is synthetic lethal with temperature-sensitive *cdc2 *(*pol δ*) mutations and with the 3'-5' exonuclease minus Pol δ mutant *pol3-01*, suggesting that Pol ε is required primarily for maintenance of genome integrity and that the DNA polymerase activity of Pol ε might be substituted by the remaining polymerase(s), if the polymerase domains are completely missing [16-18]. However, the requirement of xPol ε holoenzyme for chromosomal DNA replication in *Xenopus *egg extracts is much more strict than that of yeasts and Pol εΔCat holoenzyme cannot restore DNA synthesis activity of Pol ε-depleted extracts to the levels of mock-depleted extracts (Fig. 4) and residual DNA synthesis observed in Pol ε-depleted extracts is not authentic chromosomal DNA replication, unlike yeast systems [15-18,27]. Therefore, we conclude that the DNA polymerase activity of Pol ε holoenzyme is strictly required for normal chromosomal DNA replication in higher eukaryotes, including *Xenopus*, but it may not be absolutely required for chromosomal DNA replication in *S. cerevisiae *and *S. pombe*, which have a relative small chromosomal DNA.

If the above analysis is correct, then it is important to determine whether Pol ε holoenzyme participates in leading or lagging strand DNA synthesis. Although the present study does not answer this question, previous studies are consistent with a role for Pol ε in leading strand DNA synthesis [23]. Furthermore, Cdc45p and GINS associate directly with Mcm2–7p, a DNA helicase that works at the replication fork, in yeast, *Xenopus*, and *Drosophila *[33-35], and xPol ε also associates directly with xGINS, xCdc45p, and xCut5p, as shown in this study. xGINS, like yeast GINS, also stimulates DNA synthesis catalyzed by xPol ε. Thus, it is very likely that the DNA polymerase activity of xPol ε holoenzyme, plus xCdc45p, xGINS and xMcm2–7p, perform a coordinated function at the replication fork in *Xenopus *egg extracts. We propose that this function is required for leading strand DNA synthesis [32], while lagging strand DNA synthesis in *Xenopus *may be carried out by Pol δ, PCNA, RF-C and Pol α-primase as well as Fen1p, Dna2p, RPA, DNA ligase and RNase H [4,5,36].

## Conclusion

This work shows that the DNA polymerase activity proficient xPol ε holoenzyme complements DNA replication defect of the Pol ε-depleted *Xenopus *egg extracts. However, neither the DNA polymerase-domain-deleted xPol ε, DNA polymerase-dead-xPol ε, the catalytic subunit of Pol ε nor other subunits of Pol ε without the catalytic subunit complements the defect at all. Furthermore, we show that xPol ε holoenzyme directly interacts with xCdc45, xCut5, and xGINS, which are required for the initiation of chromosomal DNA replication in *Xenopus *egg extracts. These results are the first, direct and biochemical evidence that the DNA polymerase activity of xPol ε holoenzyme is absolutely required for chromosomal DNA replication in higher eukaryotes, unlike in yeasts.

## Methods

### cDNA cloning

The cDNA for the p260 subunit of *Xenopus laevis *Pol ε (GenBank accession no. AB259046) was isolated by 5'-RACE using *Xenopus laevis *ovary mRNA as templates and oligonucleotides primers synthesized based on the nucleotide sequence information of a partial cDNA for the p260 subunit of *Xenopus *Pol ε [24]. Both strands of its cDNA insert were sequenced with the use of an Applied Biosystems Prism dye terminator cycle sequencing kit and a DNA sequencer (ABI377). The initiation methionine was postulated on the basis of a comparison with the amino acid sequence of HeLa Pol ε p260 (gi:62198237). For cloning of the p17 subunit of XPol ε, the IMAGE cDNA clone 4783571(5') (GenBank ACC# BI349483) was obtained and sequenced. This clone did not contain a complete cDNA insert, so the 3' portion of p17 cDNA was cloned by 3' RACE using *Xenopus *mRNA prepared from *Xenopus *ovary (GenBank ACC# AB259047). For cloning of the p12 subunit of XPol ε, the cDNA clone PBX0037B02(5') (GenBank ACC# AW635703) was obtained and sequenced (GenBank ACC# AB259048).

### Rapid amplification of cDNA ends (RACE)

*Xenopus *ovary mRNA was prepared as published [37], and was used for cloning of the 3'-untranslated region by using 3'-Full RACE Core Set (TAKARA, Japan). The PCR products were cloned into pBR322-based plasmid DNA and sequenced. The 5'-terminal sequence was obtained by 5'-Full RACE Core Set (TAKARA, Japan) using *Xenopus *ovary mRNA as templates.

### Antibodies

Rabbit anti-*Xenopus *Pol ε p60 antibodies were affinity-purified as published [22] and used for making antigen-immobilized Affi-Gel 15 (Bio-Rad). The purified p60 antibodies or whole rabbit IgG (Pierce) as a control was crosslinked to Affi-Prep Protein A matrix (Bio-Rad) (1 mg of IgG per ml of matrix) and used for immunoprecipitation and immunodepletion experiments. Rabbit anti-*Xenopus *Pol ε p12 and p17 antibodies were raised by using p12 and p17, which were expressed in *E. coli *and purified, as antigens (K. Shikata, unpublished).

### Xenopus egg extracts and DNA replication assay

*Xenopus laevis *egg extracts (low-speed supernatant) were prepared as described previously [22]. Immunodepletion was performed by mixing egg extracts three times with the antibody-crosslinked matrix at 4°C. DNA replication with membrane-removed sperm nuclei (2,000 sperm heads per ml of extract) was carried out at 23°C in the presence of [α-^32^P]dATP as described [22]. The reaction products were purified by RNase A digestion, proteinase K digestion, and phenol-chloroform extraction followed by ethanol precipitation and then separated by 0.8% agarose gel electrophoresis under neutral (Tris/borate/EDTA buffer) condition as described previously [22]. After electrophoresis, the gel was fixed, dried, and subjected to autoradiography. The quantification of replication products was carried out with a Fuji image analyzer (BAS1500).

### Expression, purification, and reconstitution of Xenopus Pol ε Complex

For expression of proteins, 150 × 10^6 ^log phase sf21 insect cells were grown in suspension tissue culture flasks. The cells were infected with viral supernatant at a multiplicity of infection of 10 for p260 (a catalytic subunit of xPol ε complex), 5 for p60 (the second subunit of xPol ε complex), 5 for p12 (the third subunit of XPol ε complex), and 5 for p17 (the fourth subunit of xPol ε complex). After a 2-day incubation at 27 °C, cells were harvested, washed once with cold 1 × Tris-buffered saline, flash frozen in liquid N_2_, and stored at -80 °C. The frozen cells were thawed once on ice and then resuspended in 5 ml of buffer A (50 mM Tris, 150 mM NaCl, 5 mM EDTA, 20% glycerol, pH 7.5) containing 20 μg/ml leupeptin. After lysis, cells were kept on ice for 15 min and centrifuged at 12,000 rpm in a microcentrifuge for 10 min, and supernatant was recovered. Pol ε complex was purified from insect cell extracts with DEAE sepharose column, followed by anti-FLAG antibody column. Native *Xenopus *Pol ε (n-xPol ε) was purified from *Xenopus *egg extract by 4 sequential column chromatographies as published [22]. Throughout the purification, column fractions were assayed for DNA polymerase activity with the use of [α-^32^P]dTTP and oligo(dT)_10_/poly(dA)_400 _(1:19; 0.04 mM nucleotides) as a primer/template and were analyzed by Western blotting. One unit of Pol ε supported the incorporation of 1 nmol of dTMP under the conditions described above.

### Preparation of Pol ε-depleted Xenopus egg extracts

Immunodepletion of xPol ε was performed by mixing *Xenopus *egg extracts three times with the xPol ε-p60-antibody-crosslinked matrix [22] at 4°C as published [22,23], resulting in successful removal of more than 99% of xPol ε holoenzyme including the catalytic subunit p260 as prviously [22]. Various amounts of the n-xPol ε obtained from *Xenopus *egg extracts or r-xPol ε holoenzyme purified from insect cells and the buffer were added back to Pol ε- or mock-depleted egg extracts as published before [22]. Then DNA replication reaction was initiated by the addition of membrane-removed sperm nuclei (2,000 sperm heads per μl of extract. After incubation at 25°C for various times, aliquots were withdrawn and analyzed by neutral agarose gel electrophoresis followed by autoradiography as published before [22,23].

### Recombinant Xenopus GINS, Cut5p, and Cdc45p

6 × His-tagged Cut5p, 6 × Flag-tagged Cut5p, 6 × Flag-tagged Cdc45p, 6 × Flag-tagged Psf2p containing GINS were expressed and purified from insect cells as described previously [24,27,31].

## Authors' contributions

KS carried out the molecular biological and genetic studies, participated in the sequence alignment and drafted the manuscript. TSM carried out the purification of Xenopus proteins from insect cells and immunoprecipitation assays. YO participated in preparation of *Xenopus *egg extracts. SW participated in the design of the study and discussion. AS conceived of the study, participated in its design and coordination, performed some experiments, and helped to draft the manuscript. All authors read and approved the final manuscript.

## Supplementary Material

Additional file 1 **Cloning strategy of *Xenopus *Pol ε p260 subunit cDNA by 5' RACE**. The 6,855 bp open reading frame of *Xenopus *Pol ε p260 is shown as a white arrow (top). Red bar shows the region, where Mimura et al. previously cloned and sequenced (4,884 bp)[24]. The yellow (1,011 bp) and green (960 bp) bars represent the regions obtained by the first and second RACE, respectively. The black arrows represent primers used in RACE. E and S represent *Eco*RI and *Sph*I sites, respectively. Click here for file

Additional file 2 **Deduced amino acid sequences of *Xenopus *and human Pol ε p260**. Deduced amino acid sequences of *Xenopus *and human Pol ε p260 [38] were aligned using a ClustalW program. The identical amino acids between *Xenopus *and human Pol ε p260 are boxed. The amino acid sequence of *Xenopus *p260 is 81% identical to that of human p260. Shaded, and shaded- and blacked squired amino acids represent similar and identical amino acid, respectively. Red arrow indicates the portion of cDNA previously cloned [24]. Click here for file

Additional file 3 **Structural similarity between *Xenopus *and *S. cerevisiae *Pol ε catalytic subunit**. In the figure, DNA polymerase catalytic domains (shown by red box), the 3'-5' exonuclease domains (shown by yellow box), zinc finger domain (shown by green box), and putative nuclear localization signals (shown by blue boxes), which are missing in *S. cerevisiae *Pol ε gene (*POL2*)[6], are shown. The numbers shown in the middle of two genes are the homology, suggesting that the catalytic domain is well conserved throughout evolution. Click here for file

Additional file 4 **Cloning of *Xenopus *Pol ε p17 and p12 subunits**. (A) Amino acid sequence comparison between human Pol ε p12 and its *Xenopus *homologue. *Xenopus *Pol ε p12 consists of 116 amino acids (predicted molecular weight is about 12 kDa) and the amino acid sequence exhibits 60% identity to that of human p12 [26]. (B) Amino acid sequence comparison between human Pol ε p17 and its *Xenopus *homologue. *Xenopus *Pol ε p17 consists of 147 amino acids (about 17 kDa protein) and its amino acid sequence has 84% identity to that of human p17 [26]. The full-length cDNA for *Xenopus *Pol ε p17 was obtained by 3' RACE using the sequence of the *Xenopus *EST clone that encodes the N-terminal region of p17. Shaded amino acid indicates identical amino acid residue. Click here for file

Additional file 5 **xGINS stimulates DNA synthesis catalyzed by xPol ε holoenzyme**. DNA synthesis reactions (10 μl) contained 200 fmol ^32^P-labeled 34-mer primer/65-mer template replication substrate [32], 15 fmol r-xPol ε holoenzyme and 150 fmol (x10) xGINS, xCut5, xCdc45, or xGINS/xCut5/xCdc45. Reactions were incubated at 25°C for the indicated amount of time, terminated by addition of stop solution (5 μl), and analyzed by sequencing gel and autoradiography [32]. The ^32^P-labeled 65-mer (the reaction product) was quantified by scintillation counter. Click here for file
