# Supplementary figures and images for: The DNA polymerase activity of Pol ε holoenzyme is required for rapid and efficient chromosomal DNA replication in Xenopus egg extracts
Source: BMC Biochem. 2006 Aug 22;7:21. doi: 10.1186/1471-2091-7-21 (PMC1560149; doi:10.1186/1471-2091-7-21)

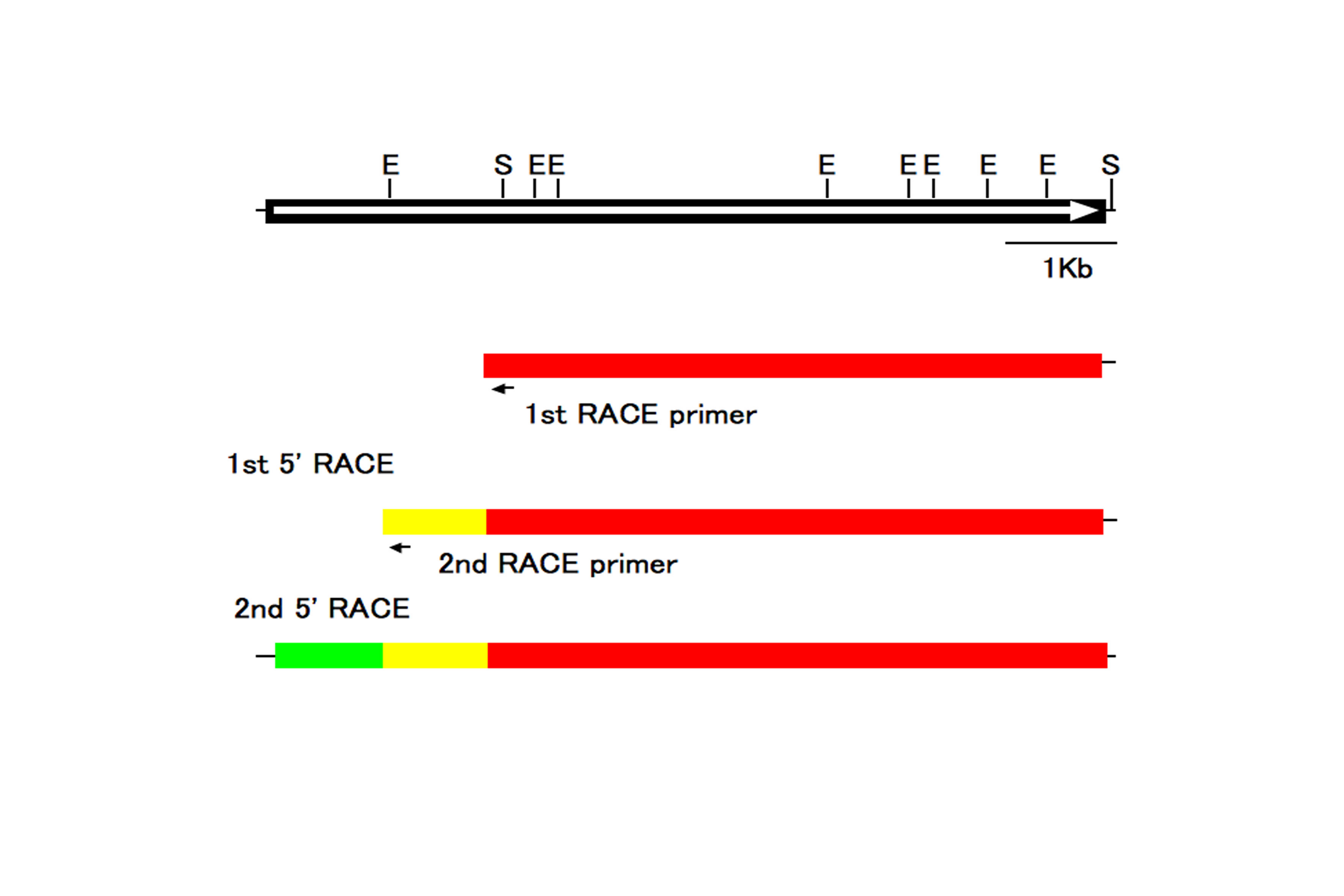

Supplement: Additional file 1 — Cloning strategy of Xenopus Pol ε p260 subunit cDNA by 5' RACE. The 6,855 bp open reading frame of Xenopus Pol ε p260 is shown as a white arrow (top). Red bar shows the region, where Mimura et al. previously cloned and sequenced (4,884 bp)[24]. The yellow (1,011 bp) and green (960 bp) bars represent the regions obtained by the first and second RACE, respectively. The black arrows represent primers used in RACE. E and S represent EcoRI and SphI sites, respectively. [file 1471-2091-7-21-S1.jpeg]

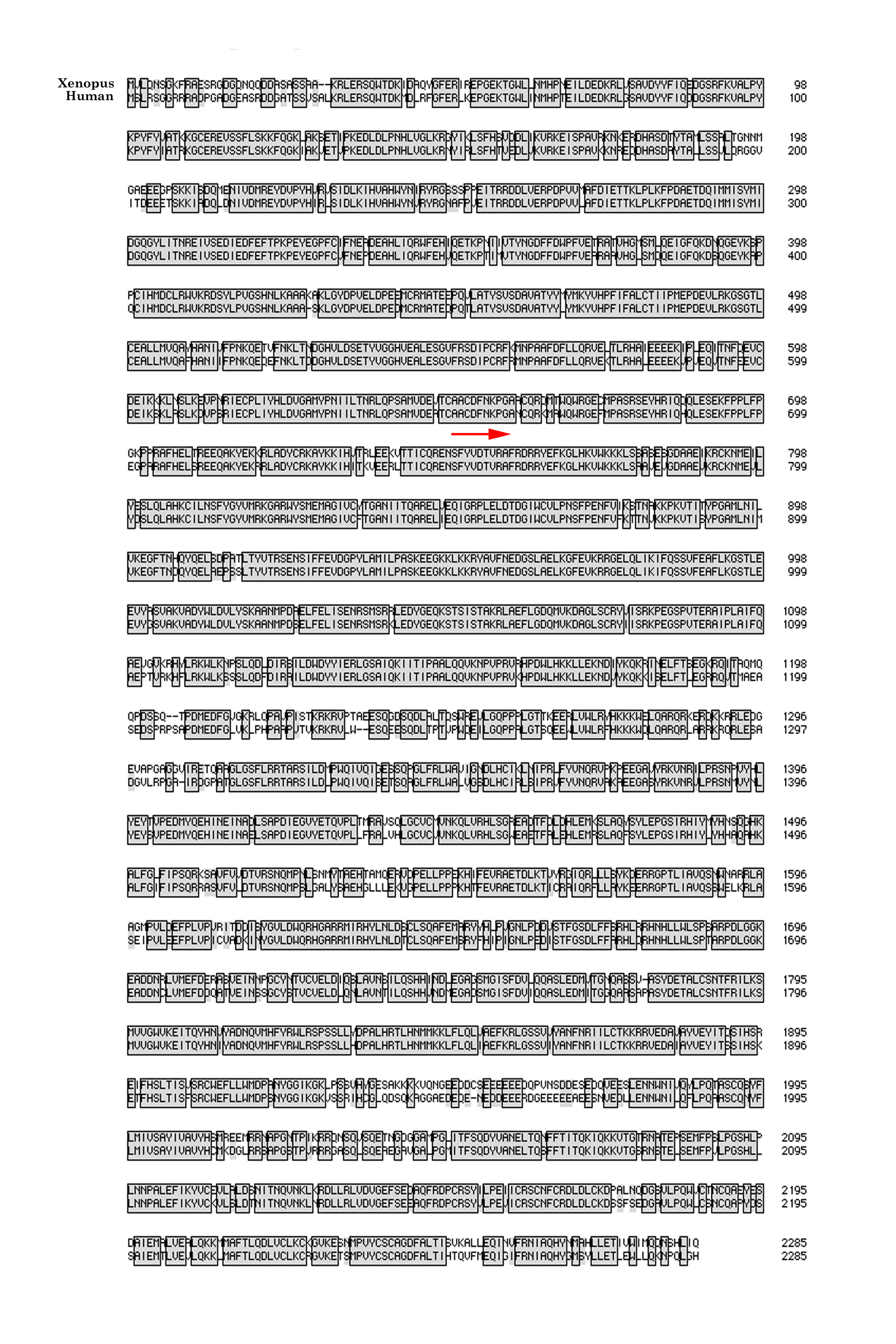

Supplement: Additional file 2 — Deduced amino acid sequences of Xenopus and human Pol ε p260. Deduced amino acid sequences of Xenopus and human Pol ε p260 [38] were aligned using a ClustalW program. The identical amino acids between Xenopus and human Pol ε p260 are boxed. The amino acid sequence of Xenopus p260 is 81% identical to that of human p260. Shaded, and shaded- and blacked squired amino acids represent similar and identical amino acid, respectively. Red arrow indicates the portion of cDNA previously cloned [24]. [file 1471-2091-7-21-S2.jpeg]

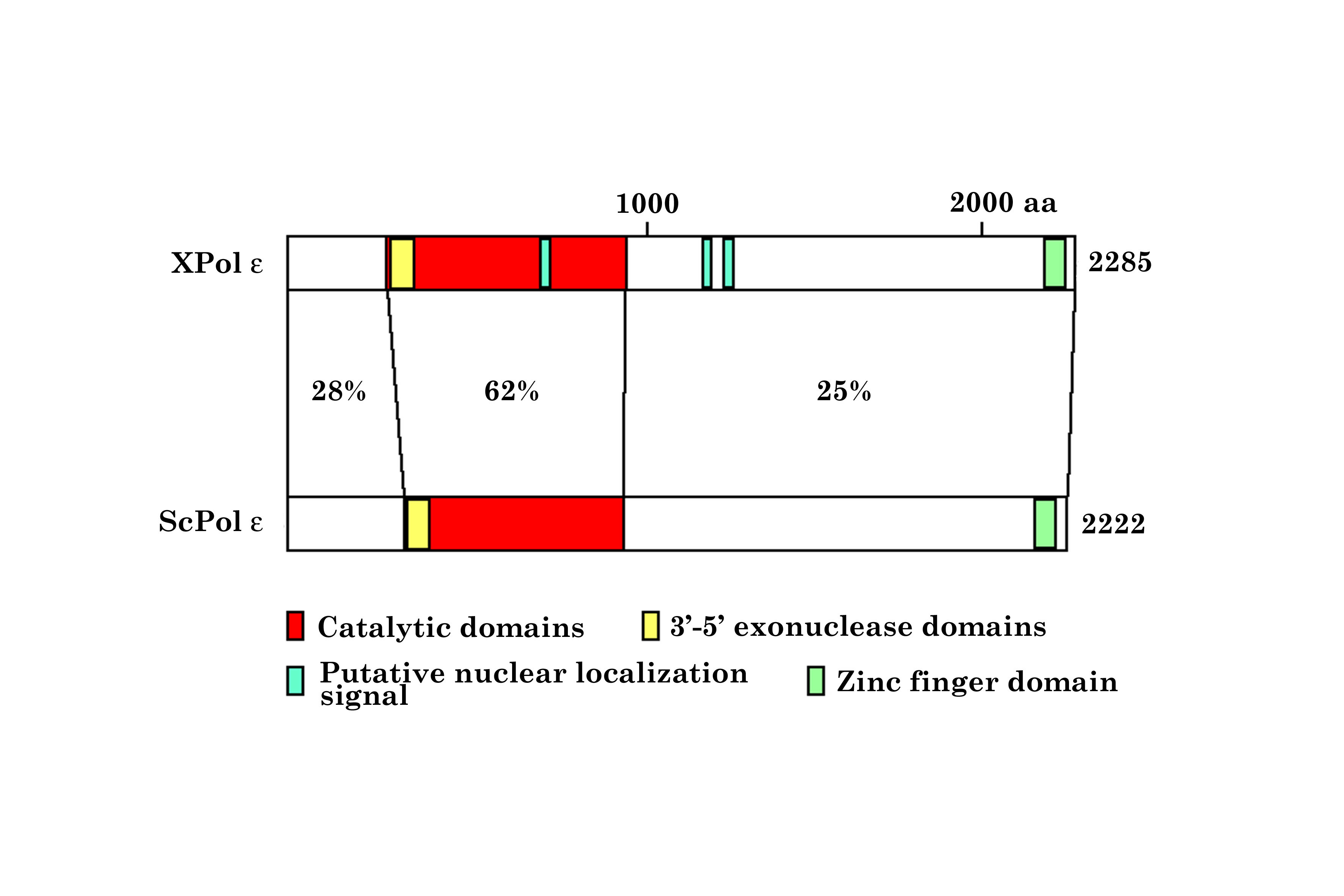

Supplement: Additional file 3 — Structural similarity between Xenopus and S. cerevisiae Pol ε catalytic subunit. In the figure, DNA polymerase catalytic domains (shown by red box), the 3'-5' exonuclease domains (shown by yellow box), zinc finger domain (shown by green box), and putative nuclear localization signals (shown by blue boxes), which are missing in S. cerevisiae Pol ε gene (POL2)[6], are shown. The numbers shown in the middle of two genes are the homology, suggesting that the catalytic domain is well conserved throughout evolution. [file 1471-2091-7-21-S3.jpeg]

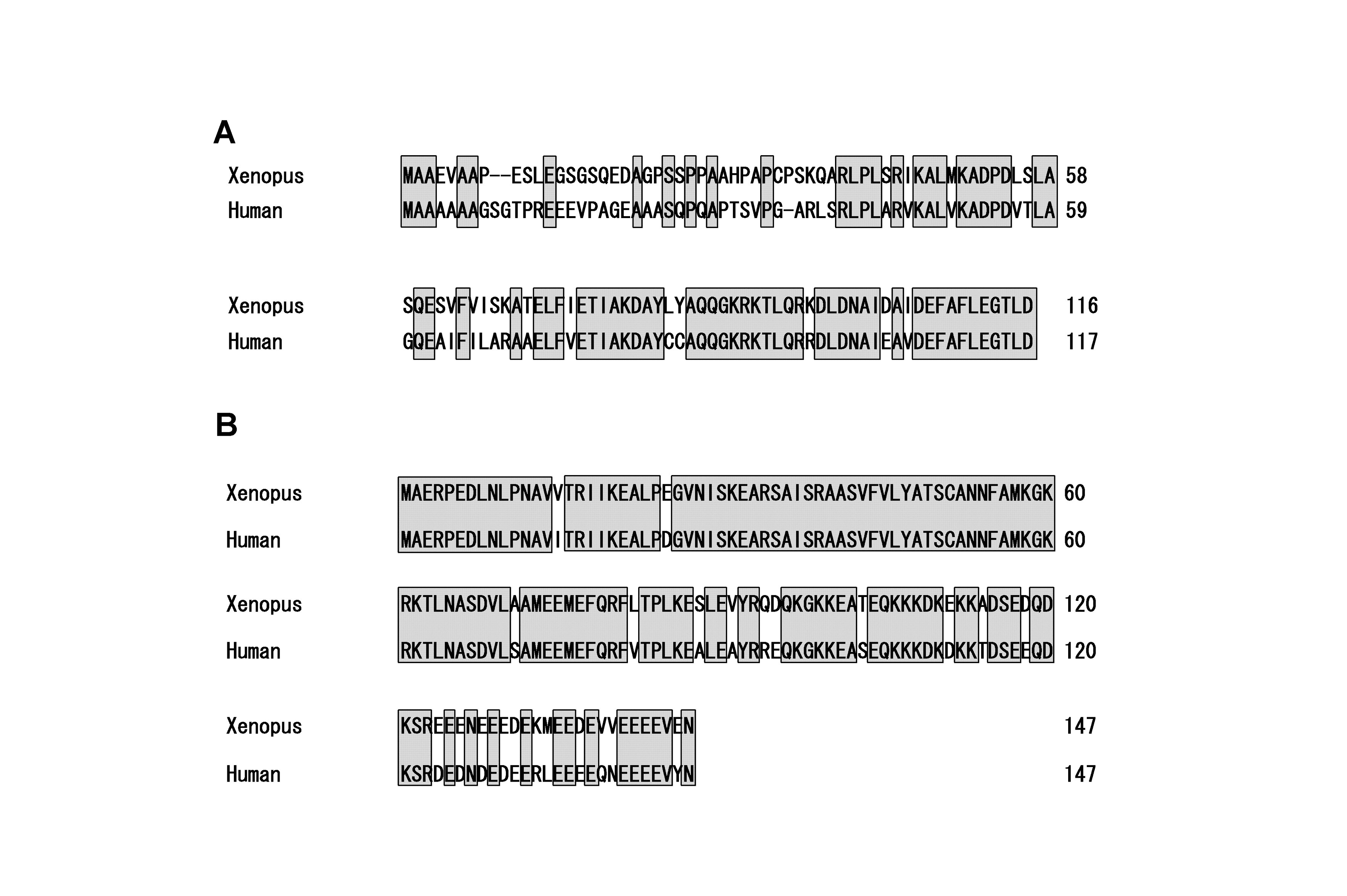

Supplement: Additional file 4 — Cloning of Xenopus Pol ε p17 and p12 subunits. (A) Amino acid sequence comparison between human Pol ε p12 and its Xenopus homologue. Xenopus Pol ε p12 consists of 116 amino acids (predicted molecular weight is about 12 kDa) and the amino acid sequence exhibits 60% identity to that of human p12 [26]. (B) Amino acid sequence comparison between human Pol ε p17 and its Xenopus homologue. Xenopus Pol ε p17 consists of 147 amino acids (about 17 kDa protein) and its amino acid sequence has 84% identity to that of human p17 [26]. The full-length cDNA for Xenopus Pol ε p17 was obtained by 3' RACE using the sequence of the Xenopus EST clone that encodes the N-terminal region of p17. Shaded amino acid indicates identical amino acid residue. [file 1471-2091-7-21-S4.jpeg]

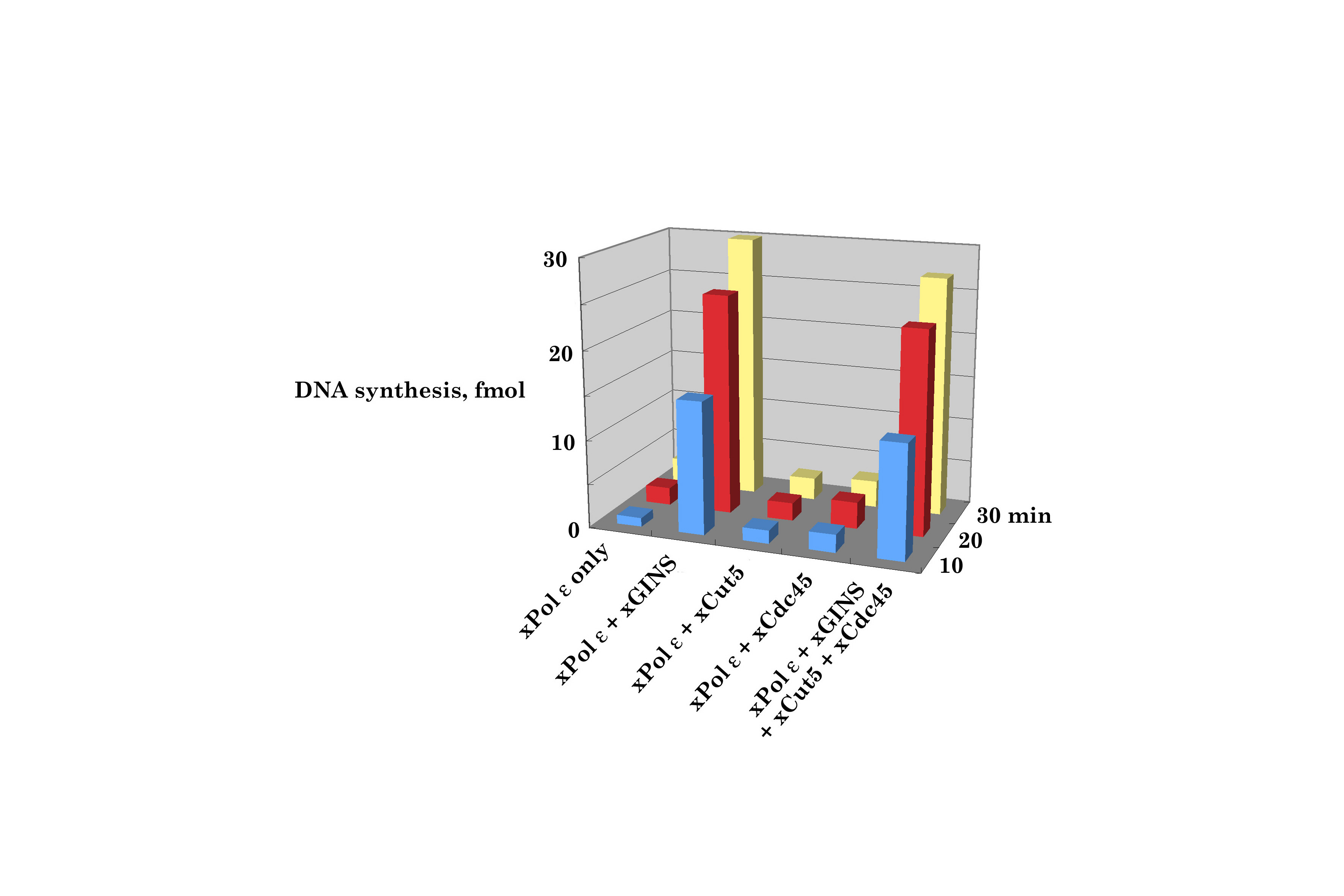

Supplement: Additional file 5 — xGINS stimulates DNA synthesis catalyzed by xPol ε holoenzyme. DNA synthesis reactions (10 μl) contained 200 fmol 32P-labeled 34-mer primer/65-mer template replication substrate [32], 15 fmol r-xPol ε holoenzyme and 150 fmol (x10) xGINS, xCut5, xCdc45, or xGINS/xCut5/xCdc45. Reactions were incubated at 25°C for the indicated amount of time, terminated by addition of stop solution (5 μl), and analyzed by sequencing gel and autoradiography [32]. The 32P-labeled 65-mer (the reaction product) was quantified by scintillation counter. [file 1471-2091-7-21-S5.jpeg]
